# Development and Validation of a Novel Prognostic Model Predicting the Atrial Fibrillation Recurrence Risk for Persistent Atrial Fibrillation Patients Treated with Nifekalant During the First Radiofrequency Catheter Ablation

**DOI:** 10.1007/s10557-022-07353-9

**Published:** 2022-06-22

**Authors:** Youzheng Dong, Zhenyu Zhai, Bo Zhu, Shucai Xiao, Yang Chen, Anxue Hou, Pengtao Zou, Zirong Xia, Jianhua Yu, Juxiang Li

**Affiliations:** Department of Cardiovascular Medicine, The Second Affiliated Hospital of Nanchang University, Nanchang, 330006 People’s Republic of China

**Keywords:** Atrial fibrillation, Nifekalant, Radiofrequency catheter ablation, Nomogram, Prognostic model

## Abstract

**Background:**

This study aimed to establish and assess a prediction model for patients with persistent atrial fibrillation (AF) treated with nifekalant during the first radiofrequency catheter ablation (RFCA).

**Methods:**

In this study, 244 patients with persistent AF from January 17, 2017 to December 14, 2017, formed the derivation cohort, and 205 patients with persistent AF from December 15, 2017 to October 28, 2018, constituted the validation cohort. The least absolute shrinkage and selection operator regression was used for variable screening and the multivariable Cox survival model for nomogram development. The accuracy and discriminative capability of this predictive model were assessed according to discrimination (area under the curve [AUC]) and calibration. Clinical practical value was evaluated using decision curve analysis.

**Results:**

Body mass index, AF duration, sex, left atrial diameter, and the different responses after nifekalant administration were identified as AF recurrence-associated factors, all of which were selected for the nomogram. In the development and validation cohorts, the AUC for predicting 1-year AF-free survival was 0.863 (95% confidence interval (CI) 0.801–0.926) and 0.855 (95% CI 0.782–0.929), respectively. The calibration curves showed satisfactory agreement between the actual AF-free survival and the nomogram prediction in the derivation and validation cohorts. In both groups, the prognostic score enabled stratifying the patients into different AF recurrence risk groups.

**Conclusions:**

This predictive nomogram can serve as a quantitative tool for estimating the 1-year AF recurrence risk for patients with persistent AF treated with nifekalant during the first RFCA.

## Introduction

Worldwide, atrial fibrillation (AF) is a common atrial tachyarrhythmia among the general population [1]. Although radiofrequency catheter ablation (RFCA) could significantly decrease the mortality risk and dramatically enhance the long-term quality of life for patients with AF [2–5], the cumulative recurrence rate has been found to be higher for patients suffering from persistent AF (PeAF) after the first RFCA with a longer follow-up [6, 7]. In the era of precision medicine, it is crucial to derivate a risk model to predict the long-term recurrence of AF after the first RFCA for patients with PeAF, which could aid in developing individualized treatment strategies. Additionally, identifying the individuals who are unlikely to maintain sinus rhythm after the procedure seems logical to increase the success probability and avoid the unnecessary risks and costs of failed ablations [8]. Several risk prediction models for AF recurrence have been developed in recent years. However, these models only moderately predict AF recurrence, and none specifically target the PeAF population [9]. Moreover, the performance of these models cannot be sufficiently assessed because the performance of model calibration was not evaluated, and the internal validation was not performed in most studies [9].

Nifekalant, a novel potassium channel blocker, has been used to treat malignant arrhythmia in the past dozen years [10, 11]. The drug exerts its antiarrhythmic effects mainly by blocking the rapid delayed rectifier K^+^ current (I_Kr_), thus prolonging the action potential duration (APD) and the effective refractory period (ERP) of atrial and ventricular myocytes [12–14]. In some AF centers, Nifekalant is already being used for immediate conversion of AF after ablation and to detect the masked critical sites of patients in whom sinus rhythm cannot be restored during RFCA [15–17]. The incidence of AF termination reported by these centers after the administration of nifekalant was approximately 65% [15–17]; moreover, the AF termination by nifekalant injection could be a predictor of higher atrial arrhythmia-free survival following the procedure [15]. Hence, a prospective cohort study was conducted to develop and validate a novel prognostic model based on different responses using nifekalant and other predictors of AF recurrence risk for 1-year after the first RFCA among patients with PeAF.

## Materials and Methods

### Patient Population and Study Design

This study was conducted at the Second Affiliated Hospital of Nanchang University (Jiangxi, China). Patients with symptomatic drug-refractory PeAF undergoing RFCA were consecutively enrolled and formed the derivation cohort between January 17, 2017 and December 14, 2017. Between December 15, 2017 and October 28, 2018, consecutive symptomatic drug-refractory patients who underwent RFCA were included in another independent data set that was then used to validate the prognostic nomogram. In these two cohorts, the final study population only included patients who were willing to accept nifekalant intravenous treatment during the operation and the patients who had previously undergone RFCA or had <1 year of follow-up after the ablation were excluded. This study was approved by the ethics committee at the Second Affiliated Hospital of Nanchang University and conducted in accordance with the Declaration of Helsinki laid down in 1964 and its later amendments. All patients provided their signed informed consent before RFCA.

### Radiofrequency Ablation Strategy

Before the procedure, electrocardiogram (ECG), 24 h Holter-ECG, 2D-echocardiography, enhanced cardiac computed tomography, and transoesophageal echocardiography (TEE) were routinely performed for all subjects. After ruling out left atrial (LA) thrombosis by TEE, low-molecular-weight heparin was used, instead of oral anticoagulation therapy, for 3 days up to 12 h before RFCA. Anti-arrhythmia agents were discontinued for ≥5 half-lives and amiodarone ≥3 months before ablation.

The ablation protocol was kept uniform for all patients. A decapolar catheter, advanced through the coronary sinus, was introduced percutaneously via the left femoral vein. Then a 20-polar circular-shaped catheter (Lasso, Biosense Webster, Diamond Bar, CA) was placed in the pulmonary veins (PVs), and a 3.5-mm irrigated-tip ablation catheter (Navistar Thermocool, Biosense Webster) was introduced by the right femoral vein. After the establishment of LA access via trans-septal puncture, heparin was administered intravenously with the activated clotting time (ACT) maintained for 300–350 s. This procedure involved the following strategy: bilateral circumferential PV isolation (PVI) was guided by using the CARTO ® system. PV antrum isolation, > 5 mm from the PV Ostia, was conducted to achieve atrial and PV bidirectional electrical conduction blockage, with the maximum power of 30–40 W, the maximal temperature of 43 °C, and an irrigation rate of 30 mL/min. The endpoint of PVI was the complete bidirectional conduction block between the left atrium and PV. After PVI, if the sinus rhythm was not achieved, nifekalant was administered intravenously at the loading dose of 0.4 mg/kg, without the continuous infusion for all patients. Different subsequent measures were performed according to different responses after using nifekalant. If the patients have converted from AF to sinus rhythm, LA substrate mapping was performed when the LA substrate was good, and the procedure was discontinued. If AF continued after nifekalant, personalized LA linear ablation was performed according to LA mapping, such as mitral isthmus, LA roof, or left anterior wall ablation. If the patients had converted from AF to atrial flutter (AFL) or atrial tachycardia (AT), activation mapping and entrainment mapping were performed, followed by targeted ablation to eliminate the AFL or AT, including mitral isthmus, cavo-tricuspid isthmus (CTI), and focal ablation.

Moreover, if typical AFL was recorded during RFCA or previously documented, ablation was conducted based on CTI. If AF was continued after the ablation or if Torsades de Pointes was observed, external electrical cardioversion was immediately administered. The different responses after nifekalant were then recorded.

The administration of oral anticoagulation was continued 6 h after the procedure, maintained for 6 months, and then stopped or continued based on the CHA2DS2-VASc score. Subcutaneous injections with low-molecular-weight heparin (1 mg/kg) every 12 h were administered if the population on vitamin K antagonists with subtherapeutic international normalized ratio (INR) until a standard INR from 2 to 3 was achieved. For all patients, after ablation, the use of AADs was determined by the clinician’s choice in accordance with the guidelines valid at that time. Normally, class 3 was recommended for patients with structural heart diseases and class 1c for general patients. AADs were used for the first 3 months and then discontinued if the patients showed no AF recurrence.

### Study Definitions and Patient Follow-up

The duration of AF was defined as the time interval between the initial onset of AF-related symptoms and the last diagnosis of AF. The primary endpoint of this study was AF recurrence, which was defined as the presence of at least one episode of AF, AFL, or AT >30 s, either symptomatic or asymptomatic on a 24 h Holter-ECG or an ECG after a blanking period of 3 months. According to the ESC guidelines for the diagnosis and management of AF [18], AF-related symptoms include palpitations, dyspnea, fatigue, chest tightness/pain, poor effort tolerance, dizziness, syncope, and disordered sleep. Outpatient follow-up visits were performed on the 1st, 3rd, 6th, 9th, and 12th months after the procedure with the 12-lead regular ECG and 24-h Holter-ECG by outpatient review. The patients were encouraged to contact the hospital immediately once they demonstrated any symptoms possibly related to AF recurrence. If a face-to-face interview was missed, a structured clinical telephonic interview was conducted. The patients who continued to take antiarrhythmic agents after 3 months of follow-up were not considered to represent ablation failure. The event-free endpoint was defined as the follow-up time of at least 1 year after the ablation.

### Sample Size

To avoid overfitting during the model establishment, we requested a minimum of 10 events per variable [19]. Based on the common rule mentioned above and 107 events, which included 64 events in the derivation cohort and 43 events in the validation cohort, we obtained an adequate sample size to consider up to six variables for candidate predictors.

### Statistical Analyses

Before the data analyses, we examined all variables’ distribution and the missing value. Although the missing value of the majority of variables identified for model development and validation was <5%, 10-fold multiple imputation by chained equations was conducted for the missing data using the R package (mice), wherein the predictive mean matching was embedded with the cases (k) = 5 default [20]. Patients who were lost to follow-up or whose demographic data were missing were excluded from the analyses.

Analyses of all data were conducted using the RStudio version 1.1.414 (Boston, MA, USA) and Empower (http://www.empowerstats.com; X&Y Solutions, Inc., Boston, MA). The continuous variables were represented as the mean ± standard deviation (SD) or median (interquartile range [IQR]), and the statistical differences were estimated using the unpaired Student’s *t* test or the Mann–Whitney U–test. The categorical variables were summarized as frequencies (percentages), and differences between 2 groups were calculated by the χ^2^ test or the Fisher’s exact test, as deemed appropriate. *P* <0.05 (two-sided) was considered to indicate statistical significance. The follow-up duration was computed from the data of undergoing the first RFCA to the data of reaching the primary study endpoint or censoring, which was defined as documented or symptomatic AF recurrence after a blanking period of 3 months detected by regular 12-lead ECG and 24-h Holter-ECG post-ablation. The Kaplan–Meier method evaluated the cumulative risks of AF recurrence as predefined in the derivation or validation cohort after a blanking period of 3 months during the follow-up.

All candidate clinical variables were determined according to the detailed literature reviews [21–26] and data availability, and the data of all variables was derived from the medical records of each subject enrolled in the study. A total of 43 candidate variables were assessed by least absolute shrinkage and selection operator (LASSO) Cox regression with 10-fold cross-validation to identify the variables of time to AF recurrence in the establishment cohort and statistically significant predictors estimated by LASSO regression; the variables considered to be informative in a clinical setting were entered simultaneously into the final multivariate Cox model [27, 28]. The Schoenfeld residuals were used to validate Cox proportional hazards regression assumptions [29]. Based on the output of the LASSO Cox algorithm, a prognostic nomogram predicting AF recurrence risk for 1-year after the first RFCA among PeAF patients was accordingly developed. The following variables were assessed: sex, age, body mass index (BMI), the duration of AF history, hypertension, diabetes, cigarette smoking, coronary heart disease (CHD), heart failure (HF), obstructive sleep apnea-hypopnea syndrome (OSAHS), cerebral vascular disease, chronic obstructive pulmonary diseases (COPD), valvular heart disease, peripheral arterial disease, congenital heart disease, hypertrophic cardiomyopathy, dilated cardiomyopathy, arrhythmic cardiomyopathy, hyperthyroidism, serum biochemical parameters, LA diameter (LAD), left ventricular ejection fraction (LVEF), ECG parameters, and different responses of nifekalant for AF conversion in RFCA.

We utilized the derivation group for internal validation and the validation group for external validation [30, 31]. The area under the curve (AUC) was conducted to assess the discriminative power of the survival nomogram. Generally, AUC ≥0.70 indicates a high discriminative ability of the model [32]. Calibration curves were prepared to measure the calibration abilities of the survival nomogram [33]. The calibration assessment of the model was performed with the 1000-bootstrap samples [19]. In addition, the decision curve analysis (DCA) curve was plotted to evaluate the clinical benefits of the prognostic model [34]. The derivation and validation of the novel nomogram were conducted with reference to the checklist in the Transparent Reporting of a Multivariable Prediction Model for Individual Prognosis or Diagnosis (*TRIPOD*) guideline (*TRIPOD* Checklist) [35]. Moreover, to present a range of risk estimates, all patients were classified into low- and high-risk groups based on the median of the risk score, which was calculated using a linear combination of selected variables weighted by their respective regression coefficients (β) from the Cox model.

## Results

### Characteristics of the Study Population

**Fig. 1:**
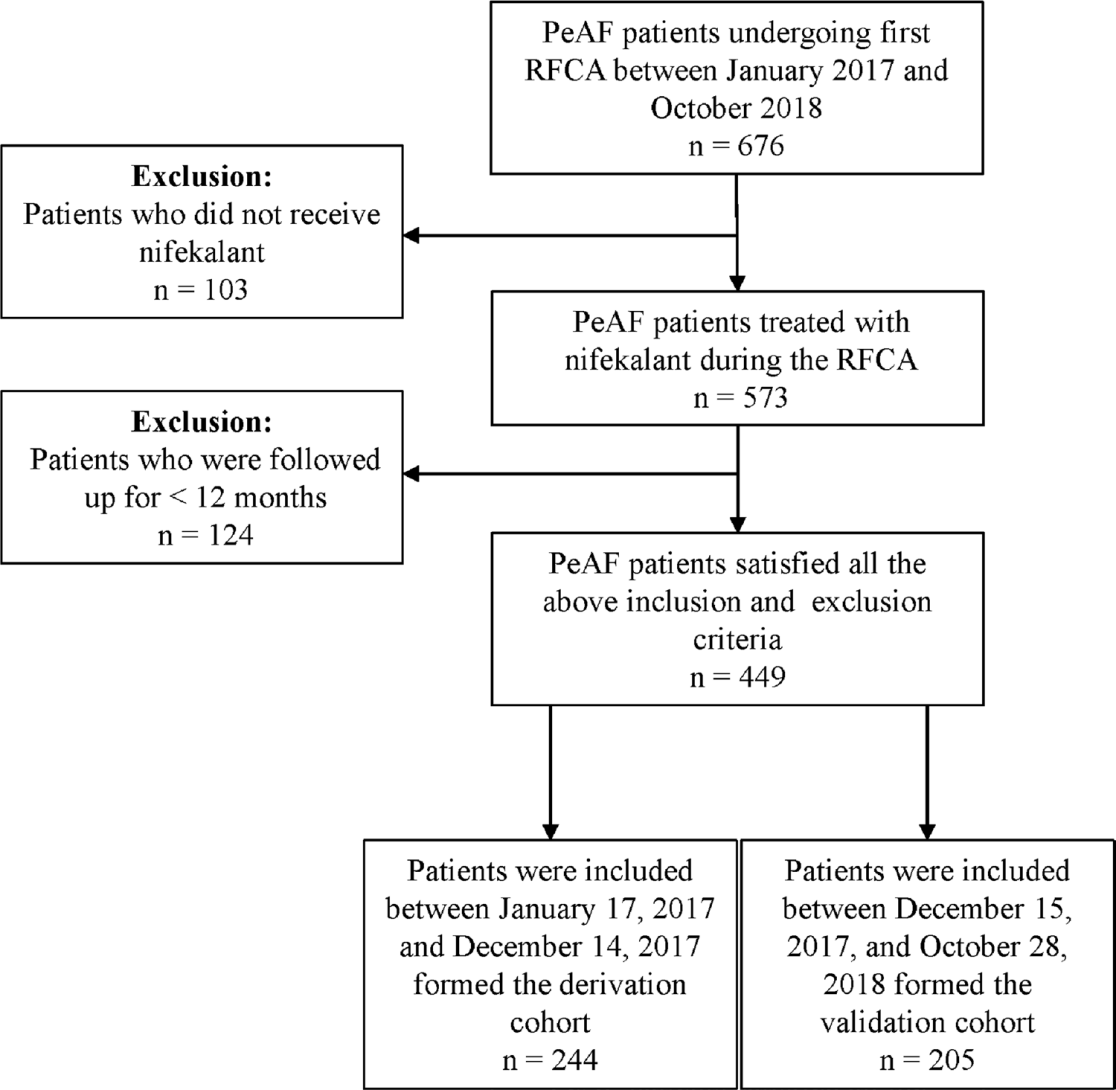
Study cohort flow diagram. *PeAF*, persistent atrial fibrillation; *RFCA*, radiofrequency catheter ablation

A total of 676 patients with PeAF were screened, of which 449 were included in the total cohort (Fig. 1). The baseline characteristics of the study population are presented in Table 1. The derivation cohort comprised 244 individuals who had undergone RFCA; their mean age was 64.16 (±9.50) years, and 150 (61.48%) were men. The validation cohort comprised 205 patients with a mean age of 64.74 (±9.24) years, of which 130 (63.41%) were men. In both cohorts, the median AF duration based on history was 12 months, and the IQR was 2.00–36.00 months in the derivation set and 2.00–44.00 months in the validation set. The derivation set presented a slightly higher level of B-natriuretic peptide than the validation cohort (development cohort: median, 188.22 (95.96–368.13) pg/mL; validation cohort: median, 174.86 (74.17–358.69) pg/mL; *P* = 0.651). However, the levels of multiple indices of renal function, including SCR, BUA, and eGFR, were slightly lower in the derivation set than in the validation cohort, without a significant difference between the two cohorts. Imaging data pertaining to indicators of cardiac function, including LVEF and LAD, were 58.34 ± 9.88% vs. 59.03 ± 9.60% and 41.31 ± 4.81 mm vs. 41.03 ± 5.40 mm in the development and validation cohorts, respectively (*P* > 0.05). The frequency of the responses after using nifekalant in RFCA for patients with PeAF, such as those with AF rhythm, AFL, or AT, was higher in the derivation cohort than in the validation cohort (AF rhythm: 60 [24.59%] vs. 38 [18.54%]; AFL or AT: 64 [26.23%] vs. 46 [22.44%]), but the frequency of the sinus rhythm after using nifekalant was comparable in the two cohorts (*P* = 0.104). The median follow-up duration (IQR) was 515 (400–773) days and 493 (422–735) days in the derivation and validation groups, respectively. Moreover, the frequency of AF recurrence after RFCA during the follow-up was 26.23% (64/244) in the derivation group and 20.98% (43/205) in the validation group, without a significant difference between the two groups (*P* = 0.193).

**Table 1:** Patients characteristics of the derivation and validation cohorts

Variables	Derivation cohort	Validation cohort	*P* value
	(*n* = 244)	(*n* = 205)	
Demographics			
Age, years	64.16 ± 9.50	64.74 ± 9.24	0.520
Male sex	150 (61.48%)	130 (63.41%)	0.673
Height, m	1.64 ± 0.09	1.63 ± 0.09	0.632
Weight, kg	67.47 ± 12.19	66.51 ± 11.21	0.391
Smoking	42 (17.21%)	36 (17.56%)	0.923
BMI, kg/m^2^	24.65 ± 3.58	25.05 ± 4.50	0.300
Previous medical history			
Duration of AF history, months	12.00 (2.00–36.00)	12.00 (2.00–44.00)	0.077
Hypertension	134 (54.92%)	98 (47.80%)	0.133
Diabetes	32 (13.11%)	20 (9.76%)	0.268
Coronary heart disease	26 (10.66%)	21 (10.24%)	0.887
Heart failure	23 (9.43%)	17 (8.29%)	0.674
Cerebral vascular disease	51 (20.90%)	37 (18.05%)	0.448
Valvular heart disease	11 (4.51%)	4 (1.95%)	0.133
Hypertrophic cardiomyopathy	7 (2.87%)	6 (2.93%)	0.971
Peripheral arteria disease	6 (2.46%)	8 (3.90%)	0.381
Dilated cardiomyopathy	26 (10.66%)	15 (7.32%)	0.221
Arrhythmic cardiomyopathy	7 (2.87%)	11 (5.37%)	0.179
Congenital heart disease	10 (4.10%)	10 (4.88%)	0.69
Hyperthyroidism	15 (6.15%)	11 (5.37%)	0.724
OSAHS	12 (4.92%)	8 (3.90%)	0.603
COPD	9 (3.69%)	10 (4.88%)	0.533
Laboratory data			
White blood cell, 10^9^/L	6.44 ± 2.24	6.66 ± 2.18	0.278
Neutrophil, 10^9^/L	4.34 ± 2.10	4.46 ± 1.98	0.553
Bloodglucose, mmol/L	5.35 ± 1.82	5.18 ± 1.16	0.236
B-natriuretic peptide, pg/ml	188.22 (95.96–368.13)	174.86 (74.17–358.69)	0.651
Homocysteine, μmol/L	13.80 ± 4.99	13.36 ± 5.04	0.354
TC, mmol/L	4.35 ± 0.99	4.41 ± 1.16	0.508
HDL-C, mmol/L	1.14 ± 0.33	1.20 ± 0.35	0.072
LDL-C, mmol/L	2.54 ± 0.77	2.52 ± 0.91	0.787
TG, mmol/L	1.51 ± 0.95	1.56 ± 1.05	0.666
SCR, μmol/L	80.80 ± 21.85	83.34 ± 25.57	0.258
BUA, μmol/L	358.16 ± 107.93	373.24 ± 117.53	0.157
eGFR, mL/min per 1.73m^2^	84.80 ± 20.68	84.96 ± 21.73	0.940
CRP, mg/dL	3.66 (1.47–10.48)	2.84 (1.30–9.33)	0.341
CA199, IU/ml	14.98 ± 10.07	15.15 ± 11.63	0.869
Imaging			
LVEF, %	58.34 ± 9.88	59.03 ± 9.60	0.454
LAD, mm	41.31 ± 4.81	41.03 ± 5.40	0.559
Preoperative QRSd, ms	93.18 ± 14.26	94.35 ± 15.66	0.418
Preoperative QTc, ms	432.35 ± 24.21	432.55 ± 31.82	0.94
Postoperative QRSd, ms	94.11 ± 15.70	94.33 ± 17.23	0.891
Postoperative QTc, ms	448.86 ± 36.11	448.20 ± 34.80	0.847
Responses after nifekalant			0.104
Sinus rhythm	120 (49.18%)	121 (59.02%)	
AF rhythm	60 (24.59%)	38 (18.54%)	
AFL or AT	64 (26.23%)	46 (22.44%)	
Procedure time, min	175.2 ± 40.0	169.5 ± 42.1	0.137
Discharge medication			0.211
None	33 (13.52%)	45 (21.95%)	
Amiodarone	167 (68.44%)	121 (59.02%)	
Amiodarone + β-blocker	24 (9.84%)	17 (8.29%)	
Propafenone	12 (4.92%)	10 (4.88%)	
Sotalol	2 (0.82%)	2 (0.98%)	
Sotalol + β-blocker	1 (0.41%)	1 (0.49%)	
β-blocker	5 (2.05%)	9 (4.39%)	
Follow-up, days	515 (400–773)	493 (422–735)	0.674
AF recurrence	64(26.23%)	43(20.89%)	0.193

Continuous data are expressed as mean ± standard deviation, or median (IQR), and dichotomous data are presented as frequency (%). *AF*, atrial fibrillation; *AFL*, atrial flutter; *AT*, atrial tachycardia; *BMI*, body mass index; *BUA*, blood uric acid; *CA*199, carbohydrate antigen 199; *CRP*, C-reactive protein; *COPD*, chronic obstructive pulmonary disease; *eGFR*, estimated glomerular filtration rate, *HDL-C*, high density lipoprotein cholesterol; *LDL-C*, low density lipoprotein cholesterol; *LVEF*, left ventricular ejection fraction; *LAD*, left atrial diameter; *OSAHS*, obstructive sleep apnea hypopnea syndrome; *QTc*, corrected QT interval; *QRSd*, QRS wave duration; *SCR*, serum creatinine; *TC*, total cholesterol; *TG*, triglyceride

### Model Development

**Fig. 2:**
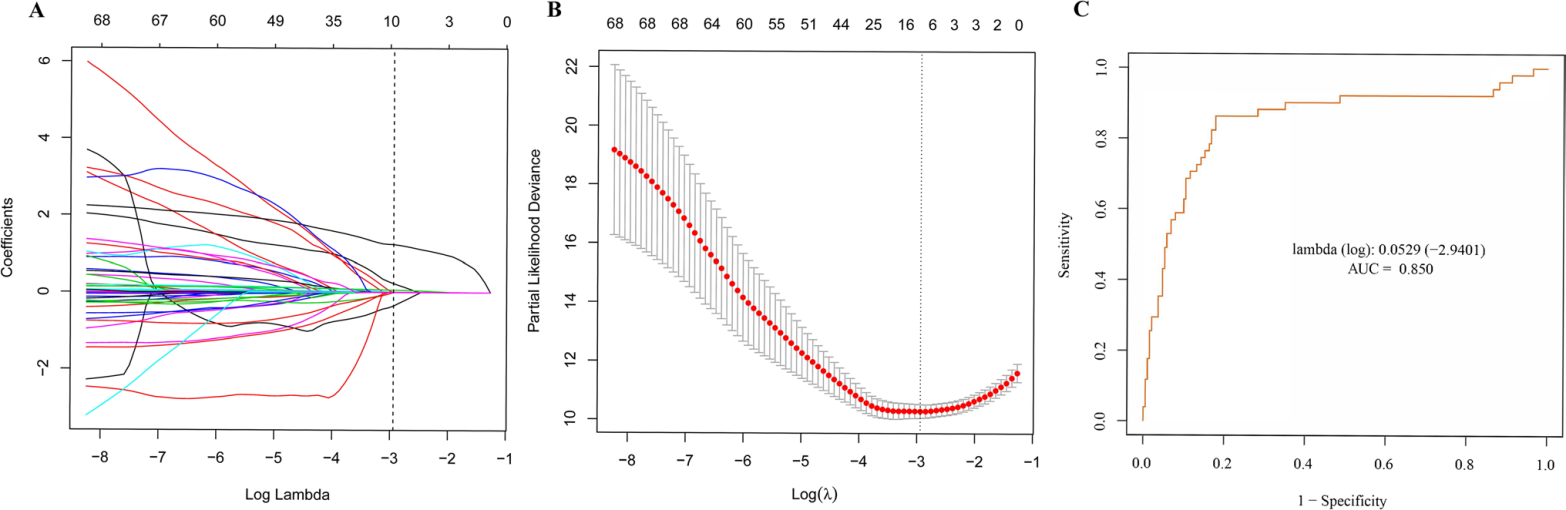
LASSO model profile plots. **A** Coefficient profile plots showing how size of the coefficients of preoperative and intraoperative factors shrinks with increasing value of the λ penalty, with the factors and their regression coefficients selected for the model based on the optimal λ for the LASSO model. **B** Penalty plot for the LASSO model; color error bars indicate standard error. **C** The optimal λ penalty of the LASSO model with a maximum AUC of 0.850. AUC indicates area under the receiver operating characteristic curve; *LASSO*, least absolute shrinkage and selection operator

**Fig. 3:**
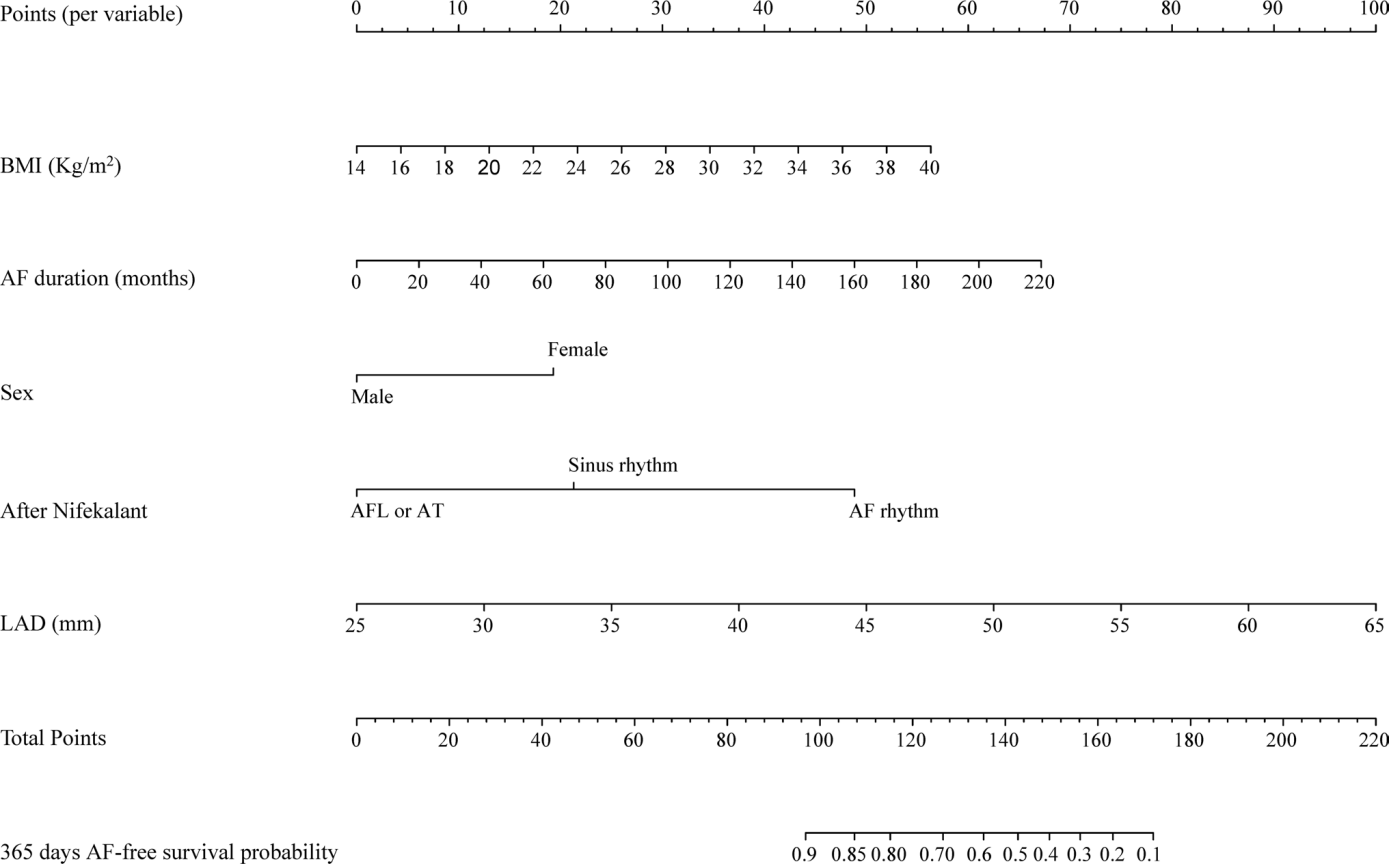
Nomogram for predicting AF-free survival probability for 365 days after RFCA. Each variable value for the individuals was determined according to the top Points scale, and then the points for each variable were added. Finally, a personalized 1-year AF-free survival probability was obtained according to the bottom Total Points scale. *AF*, atrial fibrillation; *AFL*, atrial flutter; *AT* atrial tachycardia; *BMI*, body mass index; *LAD*, left atrial diameter; *RFCA*, radiofrequency catheter ablation

**Fig. 4:**
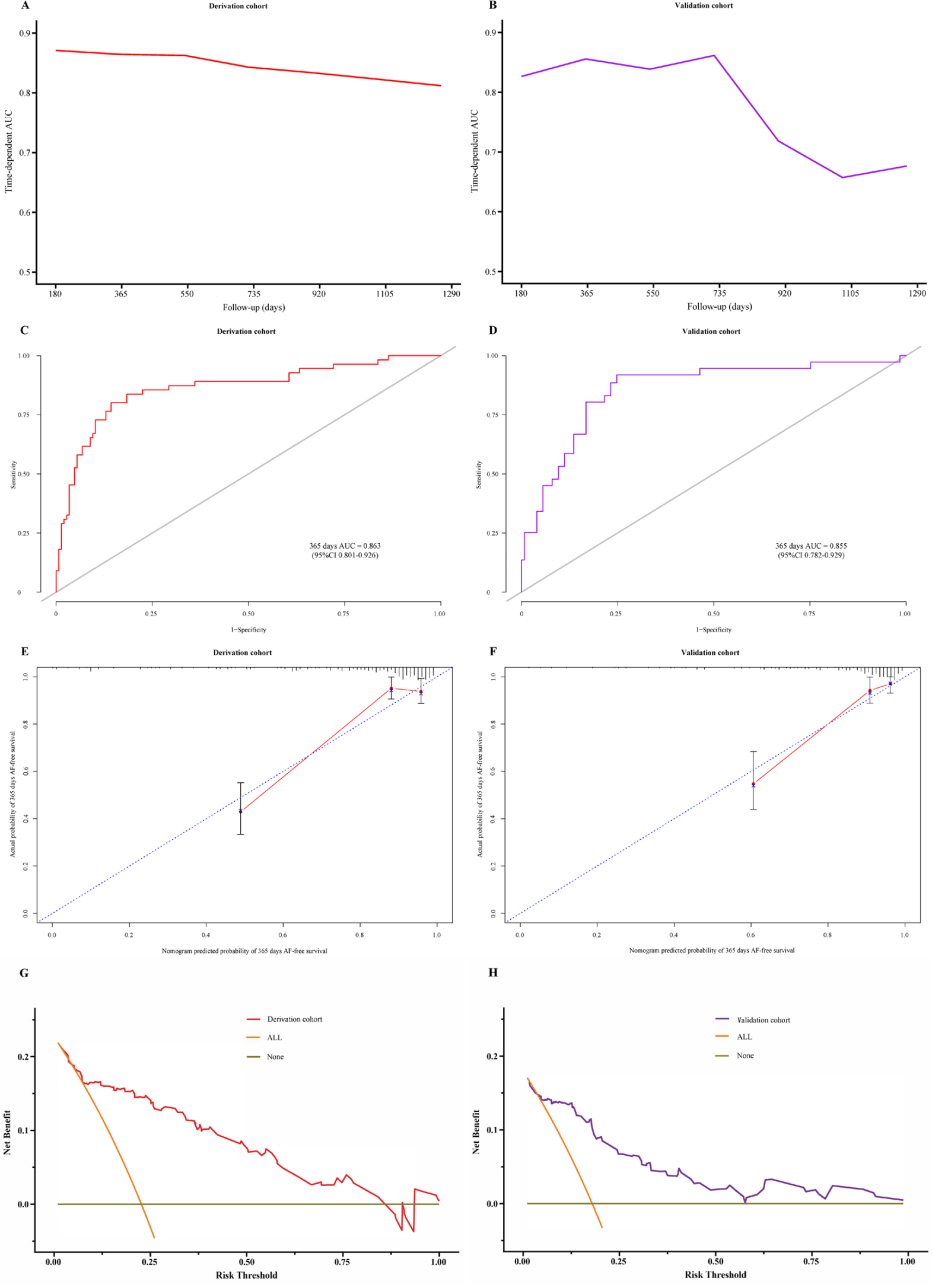
The performance of prognostic nomogram. Comparison of time-dependent AUC of derivation cohort **(A)** with validation cohort **(B)**; Comparison of 365 days AUC of derivation cohort **(C)** with validation cohort **(D)**. Calibration plots for the derivation cohort **(E)** and validation cohort **(F)** of the AF recurrence for 1-year nomogram. Decision curves for the AF recurrence for 1-year nomogram in the derivation cohort **(G)** and validation cohort **(H)**. Orange solid line indicates net benefit of a strategy of treating all patients. Gray solid line indicates net benefit of treating no patients. Color solid line indicates net benefit of a strategy of treating patients for the derivation and validation cohort according to the nomogram predictions. *AUC*, area under the curve; *AF*, atrial fibrillation

Preoperative, operative, and postoperative variables were evaluated using LASSO Cox regression analysis. This analysis identified that a panel of five predictors, namely BMI, duration of AF history, sex, responses after using nifekalant, and LAD, was most strongly associated with AF recurrence for 1-year after the first RFCA among patients suffering from PeAF in the derivation group, with the optimal λ penalty (AUC = 0.850; Fig. 2). A nomogram predicting AF recurrence for 1-year after RFCA was developed using the results from LASSO Cox regression analysis, and finally, five predictors were included in it (Table 2). The nomogram, which was used to calculate the predicted AF recurrence risk for 1-year after the first RFCA for patients with PeAF based on the multivariate Cox regression model, is shown in Fig. 3. The AF recurrence risk for 365 days after RFCA = 1 – 365 days AF-free survival probability. AF-free survival probability for 365 days can be calculated by assigning points for each predictor by drawing a line upward from the corresponding predictor to the “Points” line, summing the points, and identifying the prediction for 365 days of AF-free survival probability associated with the “Total Points” line. The time-dependent AUC based on the derivation cohort showed that the AUC value was relatively stable with increased follow-up time (Fig. 4A). The AUC for 1-year revealed that the nomogram had a higher discriminative ability, with an AUC of 0.863 (95% CI 0.801–0.926) (Fig. 4C). The calibration curve revealed a good agreement between the predicted and actual probability in the derivation cohort, which demonstrated a good fit (Fig. 4E).

**Table 2:** Multivariate Cox regression of AF recurrence risk for 365-days after the first RFCA for patients with PeAF

Variables	Hazard ratio (HR)	Lower 95%	Upper 95%	*P* value
Female	2.21	1.25	3.91	0.007
BMI	1.09	1.00	1.19	0.041
AF duration	1.01	1.01	1.02	<0.001
Responses after nifekalant				
AF rhythm	3.1	1.62	5.94	<0.001
AFL or AT	0.42	0.14	0.93	0.039
LAD	1.11	1.06	1.16	<0.001

*AF*, atrial fibrillation; *AFL*, atrial flutter; *AT*, atrial tachycardia; *BMI*, body mass index; *LAD*, left atrial diameter; *PeAF*, persistent atrial fibrillation; *RFCA*, radiofrequency catheter ablation

### Validation of the Model

External validations were used to validate the model. The time-dependent AUC based on the validation cohort showed that the AUC decreased to a certain extent with the increase in follow-up time (Fig. 4B). However, the model represented a higher performance of discrimination, with an AUC of 0.855 (95% CI 0.782–0.929) at 1 year (Fig. 4D). Calibration curves of external validations for 1-year AF recurrence estimates revealed the ideal fit of the model, where the nomogram-predicted probability (x-axis) matched the observed probability estimated using Kaplan–Meier curves (y-axis) (Fig. 4F).

### Clinical use

The decision curves of the model in the derivation and validation sets (Fig. 4G, H) presented a relatively good application value. We the threshold probability was > 0.075, the AF recurrence-predicting nomogram offered an added net benefit compared with the “all individuals with AF recurrence” or “no individuals with AF recurrence.”

### Prognostic Score for AF Recurrence Risk Stratification

**Fig. 5:**
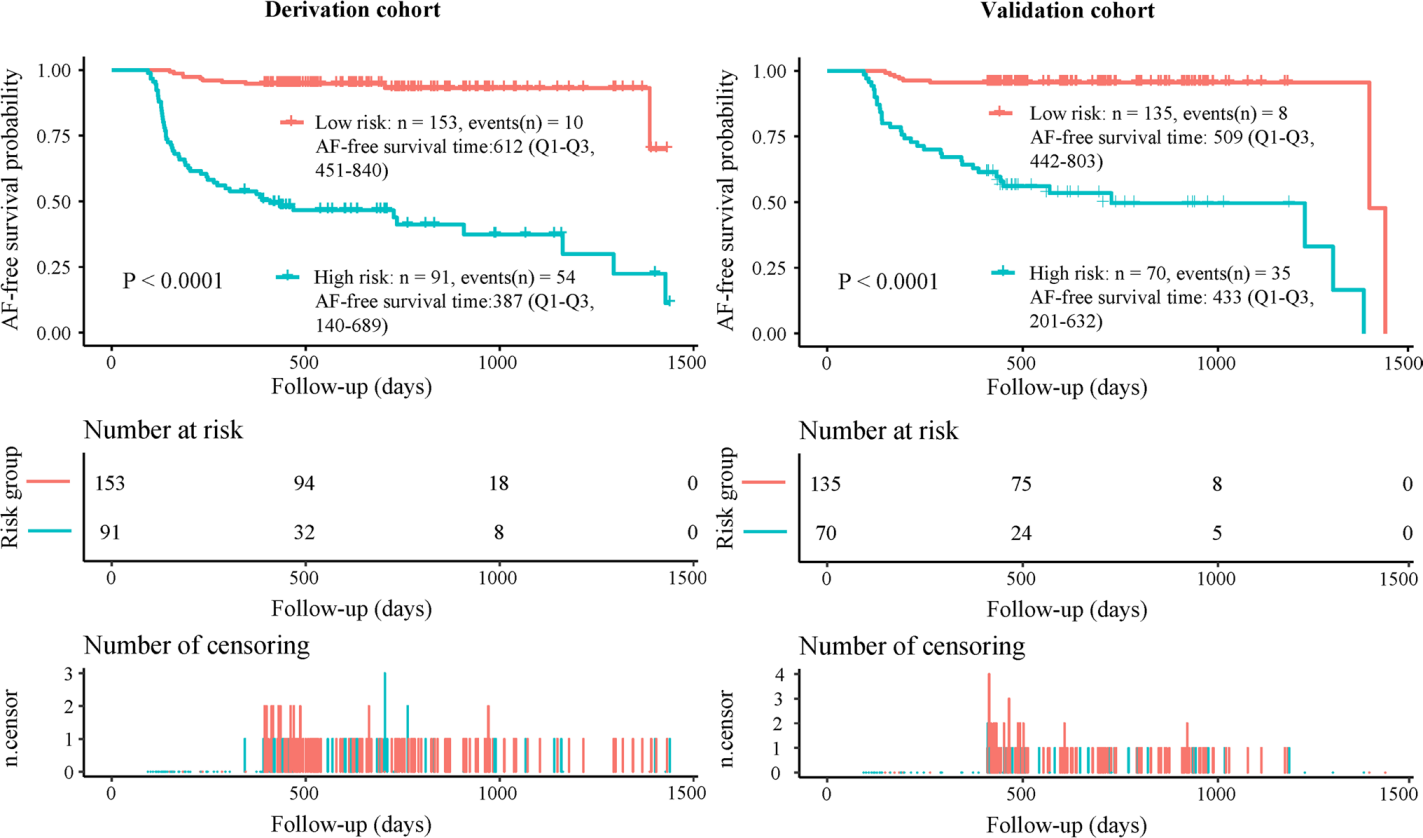
Kaplan–Meier curves of cumulative survival according to the prognostic score group for the two risk groups in the derivation cohort **(A)** and validation cohort **(B)**. Low-risk group score < 7.55; High-risk group score ≥ 7.55. Prognostic score = 0.089 * BMI (Kg/m^2^) + 0.0125 * AF duration (months) + 0.1027 * LAD (mm) + 0.7928 * (1 if sex = female) + 0 (if sex = male) + 1.1318 * (1 if after nifekalant = AF rhythm) + 0 (if after nifekalant = sinus rhythm) – 0.8752 * (1 if after nifekalant = AFL or AT). Shaded area is 95% CIs of the cumulative incidence. *AF*, atrial fibrillation; *AFL*, atrial flutter; *AT*, atrial tachycardia

Based on the final multivariate Cox regression, a prognostic score was calculated to predict the risk of time to AF recurrence after the first RFCA using a formula derived from the expression levels of these five factors weighted by their β-coefficients: Prognostic score = 0.089 * BMI (Kg/m^2^) + 0.0125 * AF duration (months) + 0.1027 * LAD (mm) + 0.7928 * (1 if sex = female) + 0 (if sex = male) + 1.1318 * (1 if after Nifekalant = AF rhythm) + 0 (if after Nifekalant = sinus rhythm) − 0.8752 * (1 if after Nifekalant = AFL or AT). Based on the median prognostic score, patients with the probability of AF recurrence for 1 year after the first RFCA were classified into two risk groups: low-risk group (*n* = 153, prognostic score <7.55) and high-risk group (*n* = 91, score ≥7.55) in the derivation cohort and low-risk group (*n* = 135, prognostic score <7.55) and high-risk group (*n* = 70, score ≥7.55) in the validation cohort. A significant difference existed between the two groups in the derivation and validation sets (*P* < 0.0001) (Fig. 5).

## Discussion

The AF recurrence risk after the first RFCA was high, and there was significant heterogeneity among the individuals [36, 37]. In this prospective cohort study, a novel prognostic model was developed and validated to generate individualized AF 1-year recurrence risk estimates for patients with PeAF after the first RFCA based on five predictors, namely BMI, duration of AF history, sex, responses after using nifekalant, and LAD. The internal and external validation results demonstrated that the predictive tool, which was presented in the form of a nomogram, had high discriminative power, with an AUC of 0.863 (95% CI 0.801–0.926). The calibration curve showed good agreement between the predicted and observed probabilities in the derivation and validation cohorts, thereby demonstrating the good fit of the nomogram. The DCA revealed that the model was a clinically available treatment algorithm associated with a high clinical net benefit for patients with PeAF after the first RFCA. Furthermore, the median prognostic score calculated using the final model aided in categorizing the patients into low-risk and high-risk groups, with a significant difference between the two groups in the derivation and validation sets (*P* < 0.0001).

Previous studies have reported many predictive variables for the recurrence of AF in patients after ablation, but the results are inconsistent [21–26]. Our analysis yielded five independent predictors associated with AF recurrence for 1-year after the first RFCA: BMI, duration of AF history, sex, responses after using nifekalant, and LAD. A meta-analysis published in 2021 showed that obesity was significantly related to a 30% increased risk of AF recurrence among patients undergoing RFCA, which agrees with our results [38]. However, in recent years, many studies have found that being overweight or obese is linked with improved poor prognosis (including death, stroke or systemic embolism and major bleeding) in patients with AF [39], a phenomenon known as the “obesity paradox.” It is unclear whether this finding is an actual physiological phenomenon or is correlated to confounding factors and needs to be clarified in the future. Duration of AF history is also considered a crucial factor associated with high clinical recurrence rates after AF catheter ablation [6, 40]. Other studies have demonstrated that sex was independently linked to AF recurrence and that there was a higher recurrence risk in women than in men, which is a universal finding [41–43]. Peng et al. reported that LAD was one of the predictors of AF recurrence after RFCA in patients with nonvalvular AF, which agrees with our findings [44]. The study by Kawaji et al. demonstrated that terminating AF via nifekalant injection during RFCA could be a clinical predictor of better success rates after the procedure [15]. This finding is consistent with some results of our study that the different outcomes of using nifekalant in RFCA were associated with the risk of AF recurrence.

In this study, after nifekalant injection, approximately 55% of patients with ablation failure were successfully converted to sinus rhythm and 25% to AT or AFL, whereas 20% of patients continued to experience AF rhythm. In multivariate Cox regression, compared with patients who converted to sinus rhythm, those who remained in AF rhythm had a substantially higher 1-year risk of AF recurrence (HR, 3.1; 95% CI 1.62–5.94; *P* < 0.001). On the contrary, those who converted to AT or AFL had a lower 1-year risk of recurrence (HR, 0.42; 95% CI 0.14–0.93; *P* = 0.039). This is an interesting finding. In our ablation protocol, for patients who converted to AT or AFL after nifekalant, targeted ablation included mitral isthmus, CTI, and focal ablation. These unmasked AT or AFL may be a key factor in maintaining AF rhythm after increasing ERP via nifekalant injection, and targeted ablation of these sites may isolate potential triggering factors and interfere with the electrical substrate of AF. However, further experimental and clinical studies are required to confirm this hypothesis.

A literature review revealed that various scoring systems have been used to predict the risk of AF recurrence after catheter ablation [45–48]. These studies included populations with all types of AF and a variety of variables. However, no predictive model has been developed specifically for patients with PeAF or for those who have used nifekalant. Moreover, these scoring systems generally had a low AUC (0.634–0.782), which suggests that they may not be able to finely discriminate the risk of AF recurrence in the ablation populations. None of the studies provided a time-dependent AUC, which is an important requirement for the long-term stability of the model. DCA, a method to calculate the clinical net benefit, is becoming an indispensable tool for model evaluation [34]. However, in these scoring systems, DCA analysis was performed in only one study with a small sample size (133) [48].

The prospective cohort study derived a novel model to predict the AF recurrence risk for 1 year after the first RFCA. The results demonstrated that the outcomes after using nifekalant were strongly associated with AF recurrence risk for 1 year after the first RFCA among patients with PeAF. The nomogram showed good discriminative power. Additionally, the calibration curve revealed good agreement between the predicted and observed probabilities in the derivation and validation cohorts, which indicates that this model has good clinical value for predicting AF recurrence in patients with PeAF after the first RFCA.

### Study Limitations

This study has several limitations. First, the recurrence rate of AF might have been underestimated because some patients with AF tend to be asymptomatic, and our follow-up protocol did not include long-term ECG monitoring. Second, although this study included many factors, some variables with predictive value were not assessed or included in the analysis (e.g., LA fibrosis, epicardial adipose tissue, and metabolic syndrome). Future studies should consider these variables. Third, this model was developed in a single-center cohort, and its external validation in several other cohorts of patients undergoing AF ablation in different centers is underway. Finally, the sample size of this study was limited, and data from multiple centers and large samples are needed to improve the model in the future.

## Conclusions

The novel prognostic model developed in this study to predict AF recurrence risk at 1-year post AF ablation based on five independent predictors, namely BMI, duration of AF history, sex, outcomes after using nifekalant, and LAD, possessed good discriminative power and calibration.

## Data Availability

All data can be obtained by contacting the corresponding author.
